# How perceived restorative environments shape meaning in life: psychological mechanisms among urban and rural active older adults

**DOI:** 10.3389/fpubh.2026.1767005

**Published:** 2026-04-20

**Authors:** Xiang Li, Yihao Huang, Donghua Yuan

**Affiliations:** School of Sociology and Humanities, Jiangxi University of Finance and Economics, Nanchang, Jiangxi, China

**Keywords:** active aging, active older adults, meaning in life, restorative environments, urban-rural differences

## Abstract

**Objectives:**

Grounded in environmental restorativeness theory, this study investigates the association between perceived restorative environments and meaning in life among active older adults. It further examines the mediating roles of psychological resilience, perceived stress, and place attachment, with particular attention to urban–rural differences in the context of population aging.

**Methods:**

A questionnaire survey collected 511 valid responses of “Active Older Adults” (aged 60–80) from some urban and rural areas of China. We employed Amos 26.0 to construct structural equation models for path analysis and mediation effect testing, and utilized SPSS 26.0 combined with the Bootstrap method to assess indirect effects. Multi-group analyses were performed to compare the structural relationships between urban and rural subsamples.

**Results:**

Perceived restorative environments were found to exert a significant direct positive effect on meaning in life (effect = 0.193, *p* < 0.001). Additionally, three indirect pathways were identified: an independent mediating effect of psychological resilience, accounting for 35.37% of the total effect; an independent mediating effect of place attachment, accounting for 25.73%; and a chained mediating effect through psychological resilience and perceived stress, accounting for 8.46%. Multi-group analyses revealed notable urban–rural differences: among urban older adults, psychological resilience played a more prominent mediating role, whereas among rural older adults, place attachment emerged as the primary pathway linking restorative environments to meaning in life.

**Conclusions:**

These findings underscore the critical role of restorative environments in enhancing meaning in life and psychological wellbeing during later adulthood. Optimizing community environments represents an effective public health strategy for promoting active aging. Age-friendly development should prioritize restorative environmental design and integrate mental health promotion, employing context-sensitive approaches tailored to the distinct needs of urban and rural older populations.

## 1 Introduction

Against the backdrop of profound global demographic shifts, aging has become an irreversible trend. The United Nations World Population Prospects 2023 report indicates that the global population aged 65 and older accounted for 9.8% in 2022, projected to rise to 16.4% by 2050 (1). China's aging process is particularly pronounced—data from the National Bureau of Statistics in 2022 revealed that by the end of 2021, the population aged 60 and above constituted 18.9% of the total population, while those aged 65 and above reached 14.2%. This significantly exceeds the 7% threshold for an aging society, signifying that China has entered a stage of deep aging (2, 3). In response to this situation, the Chinese government is systematically developing older adults resources through a three-pronged policy framework of “delayed retirement—social participation—value re-creation” (4). Both the Opinions on Strengthening Aging Work in the New Era (79) and the Decision on Implementing a Gradual Delay of the Statutory Retirement Age (80) emphasize supporting capable and healthy older adults to re-enter the labor market, enabling them to “contribute meaningfully in old age” (5, 6). This policy target group is defined as “active older adults”—a younger older adults cohort aged 60–75 with sound physical and mental functions and potential for social contribution. Their human resource development has become a core pillar of the “active aging” strategy (1, 75). This study operationalizes “active older adults” as those aged 60–80 with good self-care abilities, normal cognitive function, and a willingness or history of social participation (7, 8, 75), emphasizing their “positive state” across physiological, psychological, and social dimensions to distinguish them from conventionally defined “elderly” or “disabled” older adults.

Historically, gerontological research has predominantly focused on healthcare burdens and pathological psychological issues such as depression and anxiety. However, the positive psychology perspective underscores that old age is not merely a phase of coping with decline but a continuous process of pursuing meaning and achieving growth (9). Meaning in life, defined as an individual's perception of their existential value and life purpose, serves as a core positive factor influencing quality of life and psychological adaptation in later years (10). Research indicates that high levels of meaning in life are closely associated with better physical and mental health, higher life satisfaction, and stronger social adaptation among older adults (11). Therefore, in advancing the process of “positive aging,” how to promote active older adults to acquire and maintain abundant meaning in life has become a topic worthy of in-depth exploration.

Restorative Environments Theory offers a valuable framework for understanding the relationship between external environments and positive psychological states. This theory posits that natural and built environments possessing specific characteristics—such as richness, compatibility, and attractiveness—can enhance individuals‘ restorative experiences, thereby alleviating psychological fatigue and boosting positive emotions and cognitive function (12, 13). However, existing research on environmental restoration and older adults' mental health predominantly focuses on the broad category of “older adults,” leaving gaps in exploring environmental intervention mechanisms specifically for “active older adults.” For instance, most studies homogenize older adults, overlooking the unique needs of “active older adults” in areas like social engagement and cognitive vitality (14). Particularly from a positive psychology perspective, whether and how environments enhance meaning in life through restorative mechanisms remains under-explored. Furthermore, the “environmental perception—restorative experience—psychological benefits” pathway advocated by environmental restorativeness theory requires validation among active older adults.

Therefore, this study focuses on the group of active older adults. From the perspective of public mental health promotion, grounded in environmental restorativeness theory and guided by positive psychology, it examines the impact of perceived environmental restorativeness in communities on meaning in life. It systematically tests the mediating roles of psychological resilience, perceived stress, and place attachment in this relationship. The core question addressed is whether and how perceived restorative environment—as a modifiable environmental factor—ultimately promotes meaning in life among active older adults through specific psychological pathways. Exploring this question not only enriches interdisciplinary research at the intersection of environmental restorativeness theory and positive gerontology but also provides scientific evidence for developing evidence-based, community-environment-based mental health promotion interventions for older adults.

## 2 Literature review

### 2.1 Environmental restorativeness theory

Environmental restorativeness theory, a core branch of environmental psychology, aims to elucidate how specific environmental characteristics promote psychological recovery and positive development in individuals. This field is primarily underpinned by two theoretical frameworks: Kaplan's (12) Attention Restoration Theory (ART) and Ulrich's (15) Stress Reduction Theory. ART posits that environments possessing four key attributes—“charm, distance, expansiveness, and compatibility”—effectively repair individuals' directed attention fatigue, thereby restoring cognitive resources (16). As global aging accelerates, research focus has gradually extended to older adults. Early studies predominantly validated the general ameliorative effects of natural environments on older adults' emotions and stress (17).

Notably, existing research predominantly adopts a pathological psychology perspective of “alleviating decline,” examining how environments reduce stress, anxiety, or cognitive fatigue in older adults (18). However, from a positive psychology viewpoint, later life also represents a crucial period for pursuing meaning and realizing potential (9). Restorative environments may not only buffer negative experiences but also directly nourish individuals' meaning in life by providing immersive experiences, eliciting positive emotions, and fostering social connections (10). For the specific group of active older adults, who typically possess the capacity and willingness for sustained social engagement (75). Therefore, the significance of the environment for them may extend beyond traditional “restorative” functions, further playing a role in supporting their pursuit of “meaningful engagement in later life.” An environment with restorative qualities may indirectly provide crucial support for their quest and practice of life meaning by enhancing cognitive vitality, reducing subjective stress, and strengthening emotional attachment to activity venues (77). Consequently, this study aims to integrate restorative environment theory with positive psychology perspectives to systematically explore the underlying mechanisms through which perceived restorative environments influence active older adults' meaning in life, thereby deepening theoretical applications in the field of positive aging.

### 2.2 Perceived restorative environment

The concept of restorative environments, also termed recuperative environments, originated from the pioneering research of Kaplan and Talbot (19). Through empirical investigations of wilderness experiences, they discovered that natural settings effectively promote individual recovery from psychological fatigue and stress, introducing the core construct of “restorative environments” (19). Hartig further expanded the meaning of “recovery,” defining it as the process of renewing and enhancing physiological, psychological, and social capacities during an individual's adaptation to the environment (20). This definition transcends mere “stress relief,” suggesting the environment's potential to promote positive individual development and functional optimization.

Reexamined through the lens of positive psychology, these characteristics are not merely mechanisms for “repairing” resources but also crucial conditions for nurturing positive psychological states. For instance, “remoteness” may provide psychological space for self-reflection and value reassessment; “expansiveness” and “attractiveness” can stimulate curiosity, exploration, and immersive experiences—all foundations for generating meaning (21). While “compatibility” supports the achievement of personal goals and the smooth execution of activities, thereby enhancing feelings of control and purpose (22). Thus, perceived restorative environment is not merely about stress reduction; it may directly promote individuals' perception and construction of life meaning and purpose by providing coherent, engaging, and supportive experiences (23). For older adults, particularly active older adults, the perceived restorative environment holds uniquely significant meaning. It serves not only as a buffer against aging-related challenges but also as an enabling context for their continued growth, social engagement, and value realization. A community environment integrating natural elements and social interaction nodes not only alleviates loneliness but also provides sustained sources of meaning for older adults. This occurs by enhancing the pleasure of daily activities, fostering a sense of belonging through social connections, and cultivating emotional attachment to familiar places (24, 77). Therefore, in this study, perceived restorative environment serves as the independent variable, specifically referring to active older adults' subjective assessment of whether their environment possesses the aforementioned characteristics that promote psychological recovery and positive development. We aim to explore how this positive environmental perception ultimately enhances meaning in life through a series of psychological processes.

### 2.3 Active older adults and meaning in life

“Active older adults” refer to individuals aged 60–80 who exhibit positive physical, psychological, and social functioning (1, 7, 76). Typically possessing sound physical functioning, they can independently manage daily activities, maintain intact cognitive abilities, and exhibit curiosity toward new experiences alongside a willingness to learn. This “vitality” manifests not only in physical capacity but more profoundly in their enthusiasm for life and sense of control, enabling them to maintain high psychological resilience when confronting the challenges of aging (8). This concept overlaps with “successful aging” and “active aging” but places greater emphasis on the individual's comprehensive vitality across physiological, psychological, and social functioning (8).

Research on meaning in life among older adults is predominantly situated within positive psychology. First, numerous studies confirm the strong association between meaning in life and mental health in older adults. Those with greater meaning in life typically exhibit lower levels of depression and anxiety, along with higher subjective wellbeing. This suggests meaning in life provides psychological support, helping older adults better cope with life's stresses and difficulties (25). Research indicates that higher meaning in life correlates with lower depression and anxiety levels among older adults. Those with greater meaning in life are more likely to perceive life's positive aspects, thereby reducing negative emotional experiences (26). Furthermore, meaning in life influences older adults' coping abilities and adaptability. When confronting health issues, loss of loved ones, or shifts in social roles, older adults with a strong sense of meaning in life are more likely to adopt a positive mindset in seeking solutions (27). Meaning in life is also linked to social engagement and interaction among older adults. Research indicates that those who perceive profound meaning in life are more inclined to participate in social activities and volunteer work, thereby reducing the risk of loneliness. This active social engagement helps maintain older adults' mental health (28). In summary, meaning in life is closely associated with wellbeing, coping abilities, cognitive function, and social interaction, providing positive impacts on older adults' mental health. We hypothesize:

H1: Perceived restorative environment positively influences meaning in life among active older adults.

### 2.4 Mediating role of psychological resilience

Psychological resilience is not merely simple “recovery” after adversity but is conceptualized as a dynamic process of positive adaptation. It refers to an individual's capacity to mobilize internal and external resources to maintain or promote positive development when facing challenges, threats, or significant stressors (29). The positive psychology perspective particularly emphasizes that psychological resilience not only helps individuals withstand harm but also enables them to grow through experiences, gain insights, and deepen their understanding of life's meaning (30). For active older adults, their rich life experiences and coping expertise often constitute unique resources for developing psychological resilience. This enables them to more readily transform age-related transitions into opportunities for personal growth and meaning-seeking (31).

Placing psychological resilience within the relational framework of “environmental perception-meaning in life,” a high level of perception of restorative environments (such as experiencing environmental appeal and compatibility) may itself function as an external protective resource. By providing supportive experiences and resources, it connects with an individual's psychological resilience (e.g., enhancing a sense of control and positive emotions) (32). Second, psychological resilience, as a psychological resource, enables individuals to more proactively and effectively utilize positive elements within the environment, thereby promoting the generation and consolidation of meaning in the process. Specifically, older adults with high psychological resilience are more likely to engage in positive cognitive restructuring from daily environmental experiences (e.g., community natural landscapes, social spaces), discovering life's beauty and value. Alternatively, they may utilize the tranquility and reflective space provided by the environment to integrate life experiences and affirm life goals, thereby directly enhancing their meaning in life (11, 33). Based on this, we propose:

H2: Psychological resilience mediates the relationship between perceived restorative environment and older adults' meaning in life.

### 2.5 Mediating role of perceived stress

Perceived stress refers to an individual's cognitive evaluation and emotional response regarding whether specific relationships between themselves and their environment constitute threats, challenges, or exceed their coping resources (34). For active older adults, sources of perceived stress may include the transition to retirement, social role restructuring, changes in physical functioning, and even the digital divide (35, 36). The positive psychology perspective does not deny the existence of stress experiences but focuses more on how individuals achieve growth and meaning discovery through their interactions with stress (30). Environments with restorative qualities can effectively buffer stress responses by providing spaces “away” from daily demands, attracting non-directed attention, and supporting individuals' activity goals (12, 15). This occurs through two primary mechanisms: first, potentially reducing the perceived threat level of potential stressors; second, enhancing perceived coping resources by inducing positive emotions and relaxation responses (37). For active older adults with high social engagement and sound cognitive function, their perceived stress may exhibit heightened sensitivity to environmental characteristics, and the pathways through which restorative environments regulate their stress may demonstrate greater plasticity (38). We hypothesize that a positively perceived restorative environment can directly reduce active older adults' perceived stress levels, thereby “lightening the load” on their psychological systems. Second, this reduced stress perception creates optimized psychological conditions: it frees cognitive and emotional resources previously occupied by stress, enabling individuals to more actively pursue life goals, maintain social relationships, and engage in integrative reflection on life experiences (21). These activities constitute the core processes of constructing meaning in life (23). Therefore, the perceived restorative environment may not directly “confer” meaning but rather indirectly paves the way for individuals to autonomously construct and discover meaning by fostering a low-stress psychological backdrop. Based on this, we propose:

H3: Perceived stress mediates the relationship between perceived restorative environment and meaning in life among older adults.

### 2.6 Chain mediation effect of psychological resilience and perceived stress

Based on the preceding analysis, the promotional effect of perceived restorative environment on meaning in life may follow a chain-like progressive mediating pathway involving psychological resilience and perceived stress. This pathway integrates core perspectives from positive psychology regarding resource construction and stress transformation, transcending the isolated examination of single mediating variables. It aims to reveal the dynamic, continuous psychological processes through which environments promote positive development.

First, as a supportive external resource, perceived restorative environment can directly cultivate and enhance an individual's psychological resilience. A restorative environment characterized by attractiveness and compatibility effectively helps individuals accumulate and consolidate psychological resources (optimism, self-efficacy) by providing positive experiences (aesthetic pleasure, sense of control) and social opportunities, thereby directly elevating their psychological resilience levels (32). Second, psychological resilience significantly influences individuals' cognitive appraisal of stress and emotional responses—i.e., perceived stress. A core function of psychological resilience lies in its capacity to alter how individuals interact with stressors (29). Individuals with high psychological resilience tend to evaluate challenges in more adaptive ways and hold greater confidence in their coping resources. Consequently, they experience lower subjective stress levels when confronted with identical environmental stimuli (39). Thus, psychological resilience enhanced through environmental perception serves as a crucial internal regulator, buffering the stress induced by external demands. Finally, the reduced level of perceived stress following regulation creates favorable psychological conditions for generating and deepening meaning in life. Excessive stress consumes cognitive resources, narrows attentional focus, and hinders individuals from engaging in profound meaning-seeking and integration (21). When stress is effectively managed, individuals are more likely to shift from a “survival mode” to a “growth mode,” releasing cognitive and emotional energy to engage in activities closely linked to meaning—such as pursuing goals, deepening relationships, and self-reflection. Thus, low perceived stress serves as a vital bridge connecting psychological resources to positive outcomes (meaning in life). This hypothesized pathway elucidates, from a dynamic perspective of positive development, how restorative environments indirectly yet profoundly nurture meaning in life during later adulthood by systematically enhancing individuals' adaptive capacities and optimizing their stress experiences. This provides a more operationally feasible psychological intervention entry point for the practice of “positive aging.” This study positions psychological resilience prior to stress perception based on its theoretical status as a “core psychological resource.” Resilience is not merely a reaction to stress but an internal regulatory factor influencing how individuals assess and cope with stress (29). Therefore, this paper treats psychological resilience as an upstream variable influencing stress perception through environmental cognition, forming a progressive process of “resource gain → stress buffering → meaning construction.” This pathway aims to reflect a developmental mechanism whereby individuals gradually accumulate psychological resources through ongoing interaction with their environment, thereby optimizing stress experiences and ultimately fostering the generation of meaning. We propose:

H4. Psychological resilience and perceived stress mediate the relationship between perceived restorative environment and vitality among older adults with a sense of life meaning.

### 2.7 Mediating role of place attachment

Place attachment refers to the emotional, cognitive, and behavioral bonds formed between individuals and specific physical or social locations through sustained interaction (40). Early research often interpreted place attachment among older adults as passive dependence stemming from physical decline and reduced mobility (41, 81). However, from the perspectives of active aging and healthy aging, increasing research reveals that for active older adults in good physical and mental health, place attachment stems more from an active, constructive process (42). Through participation in community activities and cultivation of neighborhood relationships, they actively embed personal history within familiar environments, maintain social roles, and affirm self-identity. This establishes profound emotional bonds with their surroundings, transcending mere physical spatial dependence (43). From a positive psychology perspective, place attachment itself constitutes a positive psychological resource. A place to which an individual feels attached often provides a sense of security, belonging, and control—feelings that form crucial foundations for mental health and wellbeing (24). More importantly, place attachment is closely linked to the construction of meaning in life. Within this study's theoretical framework, we introduce place attachment as a variable to explore its mediating role between perceived restorative environment and meaning in life (44). Positive environmental interaction experiences are central to forming and strengthening place attachment (77). When older adults perceive an environment as relaxing, focused, and supportive, they are more likely to develop emotional closeness and identification with it. Examining this mediating pathway deepens our understanding of how environments support positive aging from the perspective of “human-environment emotional bonding.” Based on this, we propose:

H5: Place attachment mediates the relationship between perceived restorative environment and older adults' sense of life meaning.

### 2.8 Current research

In current mental health research among older adults, the sub-group of “active older adults”—who possess unique advantages in physical and mental health as well as social engagement—has long been marginalized. They are often homogenized and receive limited specialized attention, with their potential for sustained growth and value recreation through environmental influences remaining under-explored (45). Second, research perspectives remain largely confined to a pathological paradigm, focusing on exploring the mitigating effects of the environment on negative states such as depression and anxiety. Studies systematically examining how the environment functions as a positive resource to cultivate meaning in life during later adulthood from a positive psychology perspective remain insufficient (46). Finally, explorations of underlying mechanisms remain limited and superficial. The theoretical pathway of “environmental perception—regenerative experiences—psychological outcomes” has not been fully validated among active older adults, and most studies examine only single mediating variables, lacking integrated testing (47). Given these limitations, this study aims to precisely focus on active older adults. Guided by positive psychology and centering on meaning in life as the core positive outcome variable, we construct and validate an integrated model. Concurrently, we examine the chained mediating pathway of “psychological resilience—perceived stress” and the independent mediating pathway of “place attachment” to systematically reveal the operational mechanisms of perceived restorative environment. Methodologically, data will be collected via questionnaire surveys. SPSS 26.0 IBM Corporation (International Business Machines Corporation, Armonk, NY, USA) and AMOS 26.0 IBM Corporation (International Business Machines Corporation, Armonk, NY, USA) software will be employed to test the theoretical model and mediating effects through structural equation modeling, aiming to derive more robust and in-depth conclusions.

## 3 Methods

### 3.1 Research participants

Participants were selected based on the definition of “active older adults,” with inclusion criteria further specified to ensure the sample aligned with the operational definition of “active older adults.” These criteria included “ability to independently perform daily activities,” “absence of severe cognitive impairment,” and “voluntary participation in social activities.” Specifically, older adults aged 60–80 with moderate mobility were recruited from selected regions in mainland China. Prior to formal administration, a pre-survey was conducted with 30 older adults aged 60–80. Based on feedback, certain phrasing was adjusted for colloquial clarity. During the survey, trained interviewers provided one-on-one assistance with form completion, offering explanations when necessary but refraining from prompting responses. Ethical approval was obtained from the Institutional Review Board of Jiangxi University of Finance and Economics prior to study commencement. The questionnaire survey was conducted from June to August 2025 across Nanchang, Jiangxi Province, and Weinan, Shaanxi Province. A total of 532 questionnaires were collected, yielding 511 valid responses after excluding invalid data. Valid responses were distributed as 218 and 293 across the two regions, respectively, achieving a response rate of 96.05%. The sample comprised 274 males (53.4%) and 237 females (46.3%), with balanced urban-rural distribution within each region. Additional sample characteristics are detailed in Table 1.

**Table 1:** Sample characteristics distribution.

Variables	Category	Number	Percentage (%)
Gender	Male	274	53.5
	Female	237	46.3
Age group	60–65	106	20.7
	66–75	273	53.3
	75 and above	132	25.8
Region	Rural	253	49.5
	Urban	258	50.5
Monthly household income	Below 1,000 yuan	169	33.1
	1,000–3,000 yuan	107	20.9
	3,000–5,000 yuan	137	26.8
	Over 5,000 yuan	98	19.2
Living arrangements	Living alone	138	27.0
	Living with children	162	31.6
	Living with spouse	211	41.2
Education	Junior high school or below	252	49.2
	High School (Vocational School)	168	32.8
	Junior College	60	11.7
	Bachelor's degree and above	31	6.1

### 3.2 Research methods

#### 3.2.1 Perceived recovery scale

The revised Perceived Restorativeness Scale by Li et al. (48) comprises 11 items across 4 factors. Grounded in the Attention Restoration Theory, it measures individuals' perceptions of four restorative elements in the environment: distance, attraction, consistency, and scope. All items are scored using a 5-point Likert scale (1 = Strongly Disagree, 5 = Strongly Agree), with higher scores indicating greater preference for restorative environments. The Cronbach's alpha coefficient is 0.924. All items employ a 5-point Likert scale (1 = Strongly Disagree, 5 = Strongly Agree). Higher scores indicate a greater tendency toward restorative environments. The Cronbach's alpha coefficient is 0.924.

#### 3.2.2 Psychological resilience scale

The Psychological Resilience Scale developed by Connor and Davidson (49) was adopted. This comprehensive scale comprises 25 items across 5 factors, measuring positive psychological qualities that facilitate individual adaptation to adversity or coping with stressful events. All 25 items employ a 5-point Likert scale (1 = strongly disagree, 5 = strongly agree). Higher scores indicate greater psychological resilience, with a Cronbach's alpha coefficient of 0.931.

#### 3.2.3 Perceived stress scale

The revised Perceived Stress Scale by Yang and Zhang (50) was adopted. The total scale comprises 14 items across two factors, designed and measured to assess the tension and lack of control associated with stress. All 14 items employed a 5-point Likert scale (1 = Strongly disagree, 5 = Strongly agree). Higher scores indicated greater psychological stress, with a Cronbach's alpha coefficient of 0.915.

#### 3.2.4 Place dependence scale

The Local Attachment Scale developed by Williams et al. (51) and Tang et al. (52). This study optimized its combination, with the total scale comprising two dimensions: place dependence and place identification. It consists of 10 items, all scored using a 5-point Likert scale (1 = strongly disagree, 5 = strongly agree). Higher scores indicate stronger attachment to the region. The Cronbach's alpha coefficient is 0.931.

The measurement of mental health among active older adults is multidimensional. Here, the study employs the Natural Connections Scale, Social Connections Scale, Meaning in Life Scale, and Positive and Negative Emotions Scale to assess both positive and negative predictors.

#### 3.2.5 Meaning in life scale

The Chinese version of the Meaning in Life Scale adapted by Wang and Dai (53) was employed. The questionnaire comprises two dimensions: Meaning in Life Experience (“You understand the meaning of your life”) and Meaning in Life Pursuit (“You are searching for something that gives your life meaning”), totaling 10 items. All items were rated on a 5-point Likert scale (1 = Strongly disagree, 5 = Strongly agree). Higher scores indicate stronger meaning in life. The Cronbach's alpha coefficient was 0.92.

This study employed SPSS 26.0 IBM Corporation (International Business Machines Corporation, Armonk, NY, USA) for descriptive statistics and correlation analysis, and utilized Amos 26.0 IBM Corporation (International Business Machines Corporation, Armonk, NY, USA) to construct the structural equation model. The mediating effect was tested using the Process macro (Model 6) in SPSS 26.0, with 5,000 repeated bootstrap samples.

### 3.3 Research design

**Figure 1:**
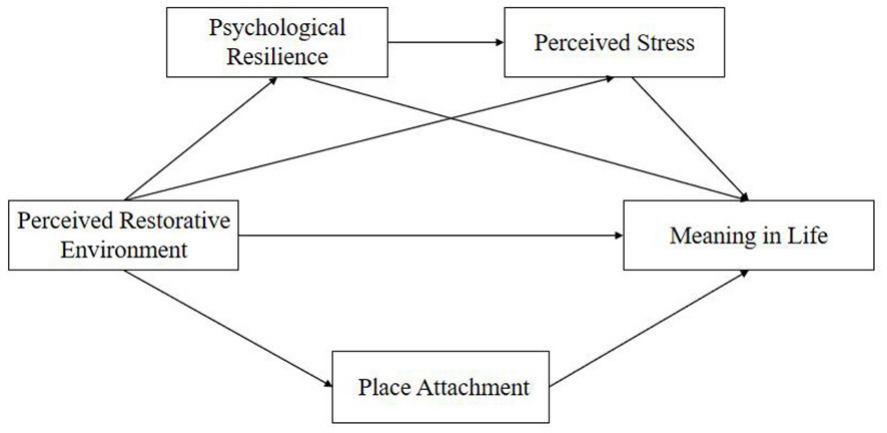
Research design diagram (conceptual model).

This study constructs a conceptual model based on environmental restorativeness theory, proposing five hypothesized pathways to examine how perceived restorative environment influences active older adults' meaning in life through mediating variables such as psychological resilience, perceived stress, and place attachment. The chained mediation model is suitable for testing mechanisms where multiple mediating variables sequentially transmit effects according to theoretical order. This model was selected because the theoretical assumption that perceived restorative environment influences meaning in life through the sequential pathway “psychological resilience → perceived stress” aligns with the analytical logic of chain mediation. Data were collected using multiple scales and analyzed via structural equation modeling (SEM) to examine path relationships and mediate effects, verifying direct and indirect interactions among variables (Figure 1).

## 4 Results

### 4.1 Common method bias test and descriptive statistics

This study employed Harman's one-factor test to examine potential common method bias in the measurement (54). Results indicated that 12 factors had eigenvalues greater than 1 prior to rotation, with the first factor explaining 34.347% of variance—below the 40% critical threshold. This confirms the absence of common method bias in the formal survey.

This study included 511 valid participants, with demographic characteristics distributed as shown in Table 1. Males (53.5%) slightly outnumbered females (46.3%) in the sample. The predominant age group was 66–75 years old (53.3%), aligning with the age definition for “active older adults.” Geographically, rural and urban samples were roughly balanced. Monthly household income exhibited a dispersed distribution, with the highest proportion (33.1%) earning less than 1,000 yuan per month. Regarding living arrangements, most older adults individuals resided with their spouse (41.2%) or children (31.6%), while 27.0% lived alone. Regarding educational attainment, nearly half of respondents had a junior high school education or below (49.2%), reflecting an overall structure dominated by secondary education levels. The above sample composition encompasses active older adults from diverse socioeconomic backgrounds, providing a representative data foundation for subsequent analysis.

### 4.2 Descriptive statistics and exploratory correlations of variables

Table 2 presents descriptive statistics for core variables. Among 511 valid samples, the mean for the independent variable Perceived Restorative Environment (PRE) was 2.87 (SD = 0.653), the mean for the mediating variable Psychological Resilience (PR) was 2.75 (SD = 0.536), the mean for the mediating variable, Perceived Stress (PS), was 3.27 (SD = 0.657), the mean for Place Attachment (PA) was 2.72 (SD = 0.663), and the mean for the dependent variable, Meaning in Life (MIL), was 2.76 (SD = 0.655). The minimum values for all variables ranged from 1.20 to 1.57, while the maximum values ranged from 4.45 to 4.60, covering the full spectrum from lower to higher levels. No significant ceiling or floor effects were observed. Notably, the mean for Subjective Perceived Stress was highest (3.27), significantly exceeding the theoretical median (3.00), while the means for Psychological Resilience and Meaning in Life were relatively low (2.75 and 2.76, respectively). This distribution pattern indicates that despite being classified as “vital seniors,” the research subjects still exhibit prominent stress management needs and room for improvement in intrinsic psychological capital and positive psychological outcomes. The variance coefficients for each variable ranged from 0.536 to 0.663, indicating moderate variability that meets the basic requirements for data distribution in subsequent structural equation modeling and mediation effect analysis.

**Table 2:** Descriptive statistics of variables.

Variables	*N*	Minimum	Maximum	Mean	Variance
PRE	511	1.36	4.45	2.872	0.653
PR	511	1.32	4.48	2.746	0.536
PS	511	1.57	4.57	3.265	0.657
PA	511	1.2	4.6	2.723	0.663
MIL	511	1.2	4.6	2.758	0.655

PRE, perceived restorative environment; MIL, meaning in life; PR, psychological resilience; PS, perceived stress; PA, place attachment.

Correlation analysis among variables revealed that perceived restorative environment (PRE) showed a significant positive correlation with meaning in life (MIL), positive correlations with the hypothesized mediating variables (psychological resilience and place attachment), and a negative correlation with perceived stress (PS). These findings preliminarily support the research hypotheses. Furthermore, meaning in life showed the strongest positive correlation with psychological resilience and a significant negative correlation with perceived stress (Table 3), laying the groundwork for subsequent testing of the chain mediation effect of psychological resilience on perceived stress (55).

**Table 3:** Correlation analysis of variables (*r*).

Variables	M	SD	PRE	PS	PR	PA	MIL
PRE	2.87	0.80	1				
PS	3.26	0.81	−0.629^**^	1			
PR	2.75	0.73	0.730^**^	−0.729^**^	1		
PA	2.72	0.81	0.643^**^	−0.661^**^	0.700^**^	1	
MIL	2.76	0.81	0.632^**^	−0.612^**^	0.700^**^	0.606^**^	1

^**^p < 0.01; PRE, Perceived Restorative Environment; MIL, Meaning in Life; PR, Psychological Resilience; PS, Perceived Stress; PA, Place Attachment.

### 4.3 Hypothesis testing and path analysis

This study employed repeated sampling of 5,000 bootstrap samples to estimate 95% confidence intervals for testing chained mediating effects. A confidence interval excluding zero indicates a significant mediating effect. The test results are presented in Table 4. Table 4 displays the model fit results for the structural equation model constructed in this study. According to commonly used standards (56, 57), all fit indices reached desirable levels: RMSEA was 0.043 (< 0.08), χ^2^/df was 1.895 (< 5), CFI, NFI, and TLI were 0.978, 0.954, and 0.973 (all >0.90), respectively, and PCFI was 0.810 (>0.50). This indicates good fit between the measurement model and data, confirming the model's validity for subsequent path analysis and hypothesis testing (58). Overall, the model demonstrated excellent fit and strong explanatory power.

**Table 4:** Structural equation model fit assessment.

Fit indices	RMSEA	χ^2^/df	CFI	NFI	TLI	PCFI
Measurement model	0.043	1.895	0.978	0.954	0.973	0.810
Fitting standard	< 0.08	< 5.00	>0.90	>0.90	>0.95	>0.50

**Figure 2:**
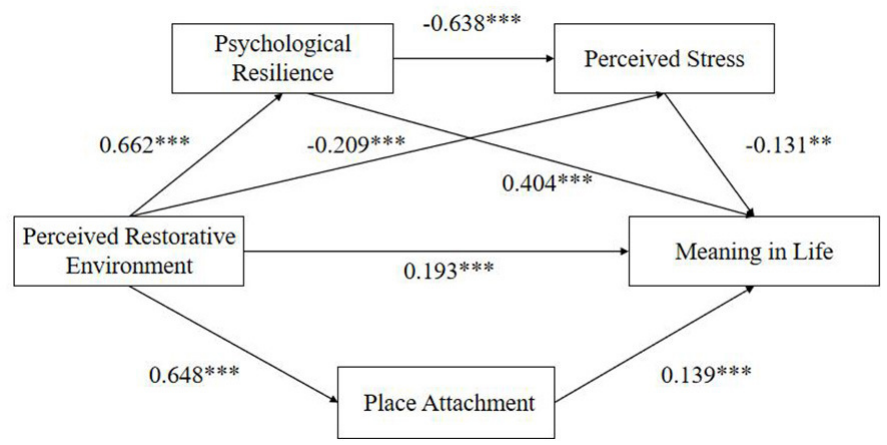
Chain mediation path.

The path analysis results of the structural equation model are shown in Figure 2 and Table 5. Perceived restorative environment significantly and positively predicted meaning in life among active older adults (β = 0.193, *p* < 0.001), confirming Research Hypothesis 1. Additionally, perceived restorative environment significantly and positively predicted psychological resilience (β = 0.662, *p* < 0.001) and place attachment (β = 0.648, *p* < 0.001), while significantly and negatively predicting perceived stress (β = −0.209, *p* < 0.001). Psychological resilience significantly negatively predicted perceived stress (β = −0.638, *p* < 0.001) and significantly positively predicted meaning in life (β = 0.404, *p* < 0.001), indicating its partial mediating role between perceived restorative environment and meaning in life, thus supporting Hypothesis 2. Perceived stress significantly negatively predicted meaning in life (β = −0.131, *p* < 0.001), indicating it also partially mediated the relationship between perceived restorative environment and meaning in life, thus supporting Hypothesis 3. Furthermore, since the path coefficients for perceived restorative environment influencing perceived stress through psychological resilience, which in turn affects meaning in life, were all significant, psychological resilience and perceived stress exerted a chain mediating effect between the two variables, confirming Hypothesis 4. Moreover, perceived restorative environment significantly and positively predicted place attachment (β = 0.648, *p* < 0.001), while place attachment significantly and positively predicted meaning in life (β = 0.139, *p* < 0.001). This result supports place attachment as an independent partial mediator between perceived restorative environment and meaning in life (59).

**Table 5:** Path tests of structural equation model.

Path	β	S.E.	C.R.	*p*	Test results
Perceived restorative environment → Psychological resilience	0.662	0.027	24.134	^***^	Established
Perceived restorative environment → perceived stress	−0.209	0.044	−4.801	^***^	Established
Perceived restorative environment → place attachment	0.648	0.034	18.968	^***^	Established
Perceived restorative environment → meaning in life	0.193	0.051	3.746	^***^	Established
Psychological resilience → perceived stress	−0.638	0.048	−13.279	^***^	Established
Psychological resilience → meaning in life	0.404	0.056	7.193	^***^	Established
Perceived stress → meaning in life	−0.131	0.045	−2.946	^**^	Established
Place attachment → meaning in life	0.139	0.039	3.569	^***^	Established

^**^p < 0.01, ^***^p < 0.001. Standardized coefficients are shown in the table.

### 4.4 Mediating effect analysis

This study employed the bias-corrected percentile bootstrap method with 5,000 bootstrap samples to test the mediating effect of perceived restorative environment on life meaning at the 95% confidence level (60). Table 6 results indicate that the total indirect effect of restorative environmental perception on the sense of life meaning among active older adults, mediated through psychological resilience, subjective stress perception, and place attachment, was 0.627. This accounted for 73.67% of the total effect (0.851), with a 95% confidence interval of [0.336, 0.480], confirming overall significant mediation. The specific indirect effects of each path are as follows: Indirect Path 1: Perceived restorative environment → Psychological resilience → Sense of life meaning [effect = 0.301, 95% CI [0.228, 0.383]], accounting for 35.37% of the total effect; Indirect Path 2: Perception of restorative environments → Perception of subjective stress → Sense of life meaning [effect = 0.035, 95% CI [0.012, 0.067], accounting for 4.11% of the total effect]; Indirect Path 3: Perceived restorative environment → Place attachment → Sense of life meaning [effect = 0.219, 95% CI [0.153, 0.284], accounting for 25.73% of the total effect]; Indirect Path 4: Perceived restorative environment → Psychological resilience → Subjective stress perception → Meaning in life [effect = 0.072, 95% CI [0.028, 0.117], accounting for 8.46% of the total effect].

**Table 6:** Mediating effects of perceived restorative environment and meaning in life.

Path	Effect	Boot, S.E.	Boot, LLCL	Boot, ULCL	*p*	Effect ratio (%)
Total path	0.851	0.034	0.566	0.701	^***^	100.00
Direct path	0.224	0.045	0.135	0.314	^***^	26.32
Total indirect path	0.627	0.036	0.336	0.480	^***^	73.67
X → M1 → Y	0.301	0.042	0.228	0.383	^***^	35.37
X → M2 → Y	0.035	0.014	0.012	0.067	^***^	4.11
X → M1 → M2 → Y	0.072	0.023	0.028	0.117	^***^	8.46
X → M3 → Y	0.219	0.034	0.153	0.284	^***^	25.73

^***^p < 0.001. X denotes perceived restorative environment, M1 denotes psychological resilience, M2 denotes perceived stress, M3 denotes place attachment, and Y denotes meaning in life.

### 4.5 Differential analysis

This study further employed independent samples *t*-tests and one-way ANOVA to examine differences in core variables—such as perceived restorative environment and meaning in life—across demographic characteristics (e.g., gender, age). Results indicate significant differences in core variables across geographic regions, with no apparent differences observed in other demographic characteristics. However, a trend emerged where higher-income groups scored higher than other groups. This suggests that core psychological variables remain relatively stable within the active older adult sample studied, showing no systematic differences based on gender or age. The influence of income on meaning in life warrants further exploration. Given this study's focus on the psychological mechanisms of environmental perception and the insignificant influence of demographic variables on mediating variables, subsequent analyses did not include gender, age, or income as control variables in the structural equation model.

Independent samples *t*-tests (Table 7) revealed significant differences between rural and urban active older adults across all core variables examined. Specifically, compared to rural older adults, urban older adults scored significantly lower on perceived restorative environment (*t* = −5.934, *p* < 0.001), psychological resilience (*t* = −4.407, *p* < 0.001), place attachment (*t* = −3.823, *p* < 0.001), and meaning in life (*t* = −3.144, *p* = 0.002). Conversely, rural older adults individuals scored significantly higher than urban older adults individuals on perceived stress (*t* = 4.097, *p* < 0.001).

**Table 7:** Independent samples *t*-test.

Variable	Rural (***N*** = 253)		Urban (***N*** = 258)		*t*	Sig (*P*)	95% Confidence interval	
	**M**	**SD**	**M**	**SD**			**Lower limit**	**Upper limit**
Perceived restorative environment	2.67	0.66	3.08	0.88	−5.934	0.000	−0.545	−0.274
Psychological resilience	2.61	0.59	2.61	0.82	−4.407	0.000	−0.404	−0.155
Perceived stress	3.41	0.69	3.12	0.89	4.097	0.000	0.150	0.427
Place attachment	2.59	0.70	2.86	0.89	−3.823	0.000	−0.411	−0.132
Meaning in life	2.66	0.71	2.87	0.88	−3.144	0.002	−0.362	−0.084

Prior to conducting the urban-rural multigroup structural equation modeling analysis, this study performed measurement invariance tests. Results indicate (see Table 8) that all fit indices for both the rural group (RMSEA = 0.047, CFI = 0.906, TLI = 0.962) and the urban group (RMSEA = 0.052, CFI = 0.954, TLI = 0.987) met the recommended fit criteria (RMSEA < 0.08, CFI > 0.90, TLI > 0.95). Combined with prior measurement invariance results (achieving scalar invariance), this confirms the comparability of measurement models across urban and rural settings, enabling subsequent multi-group path analysis.

**Table 8:** Fit assessment of the urban-rural differentiated structural equation model.

Fit indices	Region	RMSEA	χ^2^/df	CFI	NFI	TLI	PCFI
Measurement model	Rural	0.047	1.962	0.906	0.919	0.962	0.601
	Urban	0.052	1.231	0.954	0.972	0.987	0.728
Fitting standard		< 0.08	< 5.00	>0.90	>0.90	>0.95	>0.50

Based on Amos 26.0 multigroup structural equation modeling analysis, this study found that the mediating mechanism of perceived restorative environment on active older adults' meaning in life exhibits systematic differences across urban and rural settings (Table 9). For urban older adults, perceived restorative environment primarily promotes meaning in life by significantly enhancing psychological resilience [path: X → M1 → Y, β = 0.416, 95% CI [0.299, 0.544]]. Conversely, for rural older adults, this path is non-significant [β = 0.104, 95% CI [−0.006, 0.211]], with its core mechanism instead manifested through significantly enhancing place attachment [path: X → M3 → Y, β = 0.094, 95% CI [0.010, 0.194]]. This path was non-significant in urban areas [β = 0.051, 95% CI [−0.045, 0.145]]. Furthermore, the chained mediating path “psychological resilience—perceived stress” failed to reach significance in both urban and rural areas.

**Table 9:** Standardized path coefficients and confidence intervals for mediating effects across geographic groups.

Path	Rural		Urban	
	β	**95% CI**	β	**95% CI**
X → M1	0.618	[0.512, 0.718]	0.684	[0.602, 0.761]
X → M2	−0.360	[−0.515, −0.217]	−0.102	[−0.223, −0.002]
X → M3	0.585	[0.469, 0.694]	0.681	[0.586, 0.765]
M1 → Y	0.169	[−0.013, 0.353]	0.608	[0.432, 0.784]
M1 → M2	−0.418	[−0.578, −0.248]	−0.773	[−0.882, −0.642]
M2 → Y	−0.131	[−0.305, 0.025]	−0.101	[−0.250, 0.032]
M3 → Y	0.161	[0.012, 0.318]	0.075	[−0.065, 0.212]
X → M1 → Y	0.104	[−0.006, 0.211]	0.416	[0.299, 0.544]
X → M2 → Y	0.047	[−0.007, 0.122]	0.010	[−0.001, 0.044]
X → M1 → M2 → Y	0.034	[−0.003, 0.088]	0.053	[−0.016, 0.135]
X → M3 → Y	0.094	[0.010, 0.194]	0.051	[−0.045, 0.145]
Direct effect	0.310	[0.141, 0.496]	0.137	[0.029, 0.251]
Total indirect effect	0.279	[0.148, 0.409]	0.530	[0.444, 0.630]
Total effect	0.589	[0.474, 0.700]	0.667	[0.573, 0.759]

## 5 Discussion

### 5.1 The influence of perceived restorative environment on meaning in life

The findings indicate that perceived restorative environment directly and positively predicts meaning in life among active older adults. This discovery holds significant public health implications, as it identifies a measurable, intervention-eligible community environmental characteristic and establishes an empirical link between it and meaning in life—a core positive dimension of older adults' mental health. In public health, the focus is shifting from traditional disease treatment to health promotion and disease prevention, with mental health serving as the cornerstone of healthy aging (82). Meaning in life, as a key indicator of positive mental health, is not only associated with lower levels of depression and anxiety but also predicts better physical health outcomes, higher life satisfaction, and longer healthy life expectancy (61). Thus, this study extends the application of environmental restorativeness theory (12, 15) beyond its traditional scope of alleviating specific psychological symptoms (e.g., attentional fatigue, stress responses) to the public health goal of promoting overall psychological flourishing and positive aging (62, 76).

From an integrated public health and psychological perspective, this outcome demonstrates the potential of the environment as a high-level intervention point. Based on the social ecological model, individual health is embedded within multi-level environmental systems (63). The immediate environment within the microsystem represents the most accessible and relatively low-cost intervention level. The four defining characteristics of restorative environments—remoteness, expansiveness, charm, and compatibility—collectively provide daily motivation for older adults to maintain and enhance their mental health. First, “remoteness” provides psychological detachment, helping break cognitive rigidity that may arise from fixed social roles and daily trivialities among older adults. This creates opportunities for cognitive reappraisal and meaning integration, aligning with cognitive restructuring techniques used in cognitive behavioral therapy to improve mood (64). Second, “extension” and “charm” directly influence the emotional regulation system by stimulating inattentional focus and positive emotions. According to the broaden-build theory, the resulting positive emotional experiences broaden cognitive horizons, increase psychological resilience, and build enduring personal resources. These resources constitute the psychological capital needed to navigate aging challenges and sustain a sense of meaning (21). Finally, “accommodation” directly fulfills two fundamental psychological needs—autonomy and competence—as outlined in self-determination theory (22) by supporting older adults' autonomous activities. When environments enable seniors to engage in activities they deem important and valuable, their intrinsic motivation is activated and self-efficacy enhanced, forming a crucial psychological foundation for generating meaning and wellbeing. Previous research has confirmed the positive effects of restorative environments on psychological health indicators such as mood and stress among older adults (17, 18), though these studies predominantly adopted a pathological perspective focused on “alleviating negative states.” This research extends the application of this theory into the realm of positive psychology, validating a positive correlation between perceived environmental restoration and life meaning—a core indicator of positive mental health—among active older adults (β = 0.193, *p* < 0.001). The theoretical contribution of this finding lies in extending the application of environmental restorativeness theory from “repairing deficits” to “promoting flourishing.” It responds to Steger et al.'s (9) call for research on meaning in later life and provides empirical support at the environmental level for Keyes' (62) model of positive psychological wellbeing.

### 5.2 Independent mediating roles of psychological resilience, perceived stress, and place attachment

Previous studies have predominantly examined the mediating effects of psychological resilience, perceived stress, or place attachment in isolation (11, 37, 78), but few have integrated all three into a single comparative model. This study simultaneously tested all three pathways and quantified their relative contributions. The three mediating pathways identified—psychological resilience, perceived stress, and place attachment—collectively form an integrated mechanism for understanding the relationship between “perceived restorative environment” and “meaning in life.”

The mediating effect of psychological resilience was most prominent, underscoring the importance of investing in individuals' internal psychological capital for promoting mental health in older adults. Psychological resilience is not a static trait but a dynamic process that can be developed and strengthened through environmental interactions (29). Resilient environments serve as “resilience training grounds,” where each successful coping experience reinforces self-efficacy—the core of psychological resilience (65). Long-term exposure to environments featuring natural rhythms and aesthetic qualities also fosters cognitive habits of embracing change and finding positivity amid impermanence. This aligns with the goal of enhancing psychological flexibility in Acceptance and Commitment Therapy (ACT) (66). From a public health perspective, universally and unintentionally enhancing psychological resilience among community-dwelling older adults through environmental design represents a cost-effective primary prevention strategy. Psychological resilience enables older adults to better adapt to common late-life stressors such as retirement, bereavement, and health changes. It not only buffers the negative impact of these events on mental health but also facilitates “post-traumatic growth,” enabling individuals to actively construct and deepen meaning in life (30, 33). This approach is more proactive and beneficial than merely providing psychological therapy after crises occur.

The mediating role of perceived stress explains environmental influence through the lens of “reducing resistance.” This pathway draws its theoretical foundation from the cognitive appraisal model of stress and cognitive resources theory (34). Prolonged exposure to high stress constitutes a clear risk factor for various psychosomatic issues including anxiety, depression, and cardiovascular disease (67). For older adults, age-related losses of social roles, health concerns, and shifts in social support networks may constitute chronic stressors. Restorative environments, particularly those incorporating natural elements, have been shown to effectively reduce cortisol levels, heart rate, and blood pressure, inducing physiological relaxation responses (68). This rapid physiological regulation provides an immediate tool for interrupting daily stress cycles. More importantly, from a cognitive evaluation perspective, an environment perceived as restorative inherently conveys signals of “safety” and “support,” altering individuals' threat assessments of other potential stressors within the environment (34). When subjective stress levels in daily life are maintained at lower levels due to environmental support, individuals' cognitive resources are freed from persistent “vigilance-response” modes (21). This allows older adults to allocate more psychological energy toward meaningful pursuits. By reducing the average stress burden within a population, it indirectly yet broadly promotes mental health and decreases societal demand for professional services related to stress-induced psychological issues.

The mediating pathway of place attachment introduces the crucial “place” dimension from social ecology and environmental psychology. Strong social support and clear self-identity serve as vital protective factors against psychological distress and enhance wellbeing (69). Place attachment is fundamentally an emotional bond between individuals and their environment, often closely tied to maintaining social relationships and affirming personal/collective identity (43). Restorative environments, due to the positive experiences they provide, are closely associated with ideal locations for forming such emotional bonds. When older adults develop attachment to a community park, activity center, or neighborhood corner, that place often becomes a vital node in their social network. Regular visits to the same location facilitate the formation of “familiarity communities,” fostering informal social interactions and support (70). Such place-based social connections directly contribute to reducing social isolation and loneliness (71). Furthermore, place attachment reinforces older adults‘ sense of self-continuity through “place identity” (44). During identity transitions like retirement or children leaving home, a familiar place rich with personal memories helps older adults maintain a stable sense of “I am still me,” buffering identity crises stemming from role loss. From a public health practice perspective, this implies that community environment development should extend beyond providing physical facilities. It should consciously employ participatory design, community arts programs, and local history revitalization to enhance older residents' emotional investment and shared ownership of public spaces. This embeds social support and identity resources within the physical environment.

### 5.3 Chain mediation effect of psychological resilience and perceived stress

Another theoretical innovation of this study lies in its first-ever validation of the chained mediating pathway—“perceived restorative environment → psychological resilience → perceived stress → meaning in life”—among active older adults. This provides a chain hypothesis explaining how environmental influences generate long-term, deep-seated mental health benefits. This demonstrates a process from “resource accumulation” to “effective resource utilization” to “health benefits.” This finding transcends previous studies examining parallel mediation between psychological resilience and perceived stress (39), revealing a progressive relationship between the two and providing more refined mediating outcomes for public health program evaluations.

This chain model aligns with the logic of the Conservation of Resources Theory (72). In the first stage, restorative environments function as supportive resources, primarily helping individuals acquire and pursue another critical resource—psychological resilience. This constitutes a “resource gain” process. In the second stage, the acquired psychological resilience resource is mobilized to protect individuals from the loss of another resource—preventing excessive depletion of subjective stress resources through more positive stress appraisal. This constitutes a “resource conservation” process. Ultimately, when core psychological resources are secured and daily resource depletion is controlled, individuals attain a state of relative resource sufficiency, thereby gaining the capacity to pursue and experience meaning in life—a positive resource representing psychological benefits. This finding holds significant implications for public mental health practice: evaluating the effectiveness of an environmental improvement project should not focus solely on whether it affects residents' stress levels or boosts short-term mood. Instead, greater attention should be paid to whether it aligns with psychological resilience in older adults over the long term. Enhancing psychological resilience signifies the strengthening of an individual's internal protective factors, yielding more enduring and widespread health benefits that better equip individuals to cope with future challenges. Therefore, designers and evaluators of public health programs should consciously incorporate measurements of positive psychological capital, such as psychological resilience, to capture the more fundamental long-term health benefits that environmental interventions may yield. It should be noted that although this study constructed a mediation pathway model based on theory and tested it using structural equation modeling, the cross-sectional nature of the data precludes confirmation of causal relationships between variables. The identified mediating effects should be understood as statistically consistent with the theoretical model rather than definitive evidence of causal chains. Future research should further validate causal directions through longitudinal tracking or experimental designs.

### 5.4 Regional differentiation analysis

The urban-rural multi-group analysis in this study identified the dominant mediating mechanism of perceived restorative environment influencing meaning in life, revealing systematic differences between urban and rural active older adults. These differences encompass not only variations in variable means but also qualitative distinctions in the underlying mechanisms. This finding strongly refutes the simplistic tendency to view the older adults population as a homogeneous group. The urban-rural divergence revealed in this study—where psychological resilience pathways dominate in urban settings and place attachment pathways prevail in rural areas—serves as a classic illustration for contextualized interventions in public health. It powerfully underscores that any effective public health strategy must deeply understand the specific social, cultural, and physical environments in which target populations are embedded (73). This finding powerfully refutes the simplistic tendency to homogenize the older adults population and points to new directions for future research. It necessitates testing the applicability of theoretical models across diverse sociocultural contexts rather than uncritically extrapolating mechanisms identified in specific samples.

Urban environments are characterized by anonymity, high mobility, and rapid socioeconomic change (36). Older adults' social networks may be more dispersed, diverse, yet also more fragile. Their dependence on and utilization of the environment tends to be more “instrumental” and “selective.” Consequently, the impact of the environment on mental health is increasingly manifested through empowering individuals to adapt to this complexity. A restorative environment that enhances self-efficacy, offers opportunities to learn new skills, and supports the establishment of new social roles best meets their psychological needs. This, in turn, connects to a sense of life meaning through psychological resilience. Urban public health practitioners should prioritize creating diverse, accessible community environments that support individual development and active social participation.

Conversely, in rural settings, social structures tend to be relatively stable and community bonds strong, though formal public services and recreational facilities may be limited (42). The lived world of older adults is highly localized, with social identities deeply tied to specific places. The environment serves not merely as a backdrop for activities but as a vessel for social relationships, cultural traditions, and personal histories. Consequently, the impact of the environment on mental health is more directly mediated through emotional and social connections to place. Disrupting these bonds can significantly undermine the mental wellbeing of older adults (74). Public health interventions in rural areas should prioritize protecting and strengthening the existing social and physical functions of places that hold significant meaning for older adults. Improving the comfort and safety of rural public spaces, supporting community activities rooted in local cultural traditions, and encouraging older adults' participation in village affairs management—these measures can effectively connect with their place attachment, thereby solidifying their social support and sense of belonging, directly contributing to mental health.

### 5.5 Practical implications and policy recommendations

Based on the above findings, this study proposes the following targeted recommendations to enhance the sense of meaning in life for active older adults and promote positive aging practices. First, given the direct effect of “perceived restorative environments on the sense of meaning in life,” incorporate restorative qualities into urban and rural environmental construction standards. It is recommended that departments such as housing and urban-rural development, planning, and civil affairs explicitly include assessment requirements for the “restorative qualities” of spaces when formulating urban and rural environmental construction standards. Specific evaluation metrics may reference the four key characteristics of restorative environments: whether they provide quiet, secluded resting spaces (remoteness); whether they feature diverse natural elements and activity options (extensiveness and charm); and whether walkways, seating, fitness facilities, etc., are accessible for safe and comfortable use by older adults (compatibility). By “designing” mental health into the physical environment, universal, low-stigma primary prevention of mental health issues can be achieved. Second, based on insights from the three mediating pathways and their relative contributions, develop differentiated community mental health promotion programs. It is recommended that community health service centers, social work agencies, and community organizations collaborate to develop “Community Exploration and Resilience Building” programs targeting the psychological resilience pathway. Examples include organizing regular nature exploration activities for older adults to discover and utilize restorative community resources (e.g., parks, green spaces, waterfront areas), while integrating stress management training and life review workshops. This helps older adults accumulate successful coping experiences through environmental interaction, strengthening their sense of self-efficacy to cultivate psychological resilience. Additionally, develop the “Our Community Memories” program targeting the place attachment pathway. Activities like mapping community resources, recording local oral histories, and jointly maintaining community gardens or village archives enhance seniors' emotional investment and shared ownership of public spaces, transforming place attachment into observable, shareable mental health assets. Third, drawing insights from chained mediation pathways, establish a long-term impact assessment system for environmental interventions. Evaluating the effectiveness of an environmental improvement project should not focus solely on whether it immediately reduces residents' stress levels or boosts short-term mood, but should also incorporate measurement of intermediate process indicators. It is recommended to establish a multi-time-point tracking evaluation system for community environmental interventions, encompassing psychological resilience, perceived stress, and sense of life meaning. This approach captures the more fundamental long-term health benefits potentially generated by environmental interventions. For instance, measuring these indicators at project initiation, 6 months post-implementation, and 12 months post-implementation can validate the actual occurrence of dynamic processes. Fourth, drawing insights from urban-rural differences in pathway transformations, implement contextually precise intervention strategies. For urban communities, prioritize creating environments that support individual development and active social participation. Specific measures include establishing community learning centers, interest group activity rooms, and intergenerational exchange spaces; supporting seniors in establishing new social roles; developing community guide apps to help seniors discover restorative resources nearby; and organizing “Community Resilience Ambassador” training to cultivate senior leaders who can mobilize neighbors for collective participation. For rural communities, prioritize protecting and strengthening existing local social and material functions that hold significant meaning for older adults. Examples include restoring traditional social spaces like resting spots under ancient trees at village entrances or activity squares in front of ancestral halls; supporting festive events and temple fairs rooted in local traditions; and encouraging older adults to participate in village public affairs deliberation and decision-making to reinforce their identity as “village stewards.” Finally, from a health equity perspective, prioritize investments in resource-deprived communities. Public resources should be consciously directed toward rural areas, aging urban neighborhoods, and low-income communities. Implement restorative environmental improvements and mental health promotion programs first in these resource-deprived areas to ensure all older adults have equitable access to community environments that support their mental wellbeing, thereby reducing health inequalities stemming from geographic and socioeconomic disparities.

## 6 Conclusions and outlook

This study examined the relationship between perceived restorative environment and meaning in life among active older adults, exploring its potential mechanisms. Key findings include: a significant positive correlation between perceived restorative environment and meaning in life, consistent with three mediated pathways—enhanced psychological resilience, reduced perceived stress, and strengthened place attachment. Further analysis revealed that psychological resilience and perceived stress form a chained mediating pathway, consistent with the progressive process where environmental resources enhance individual traits to facilitate effective stress management. Notably, these mediating pathways exhibit significant differences between urban and rural contexts: in the urban sample, the association mediated by psychological resilience was more pronounced, while in the rural sample, the mediating effect of place attachment was relatively more significant.

The theoretical contribution of these findings lies in deepening and expanding the application of environmental restorativeness theory within geriatric mental health. They construct a multi-pathway model integrating positive psychology, health psychology, and environmental psychology, emphasizing the importance of sociocultural contexts in shaping health determinants. At the public health practice level, this study clearly points to a direction for inclusive mental health promotion that transcends traditional medical models. Specifically, it advocates for the systematic cultivation of psychological capital among older adults, the alleviation of their stress experiences, and the strengthening of their social connections and sense of belonging through the conscious planning, design, and optimization of the “restorative” qualities of community and residential environments. This approach supports the realization of “positive aging” and the construction of an “age-friendly” society.

This study has certain limitations: The cross-sectional design used cannot confirm causal directionality, only revealing associations between variables and model consistency. Future research should employ longitudinal tracking designs or multi-time-point cross-lagged analyses to more precisely examine the dynamic interaction between psychological resilience and perceived stress. Investigate whether the dominant influence direction shifts across different life events or stages, exploring the possibility of bidirectional or cyclical models. While the sample covered both urban and rural areas, it did not further subdivide geographic subtypes. The study primarily relied on self-report scales. Future research could employ longitudinal tracking or quasi-experimental designs, integrating behavioral observation, physiological indicators, and in-depth interviews to dynamically capture micro-processes of environmental interaction and meaning construction. Cross-cultural comparisons across different international and ethnic groups are also warranted. Additionally, the observed trend of income influencing life meaning was not statistically significant, potentially due to sample distribution limitations. Future research should employ larger samples, incorporate socioeconomic status variables into analysis, and explore its moderating role in the relationship between environmental perception and psychological outcomes. Simultaneously, further investigation into the moderating effects of variables such as personal interests, cultural values, and community social capital is warranted to construct a more comprehensive and contextualized theoretical model. This would provide scientific foundations for positive aging practices across diverse cultural contexts globally.

## Data Availability

The raw data supporting the conclusions of this article will be made available by the authors, without undue reservation.
